# Role of Soil, Crop Debris, and a Plant Pathogen in *Salmonella enterica* Contamination of Tomato Plants

**DOI:** 10.1371/journal.pone.0001657

**Published:** 2008-02-27

**Authors:** Jeri D. Barak, Anita S. Liang

**Affiliations:** Produce Safety and Microbiology Research Unit, Western Regional Research Center (WRRC), Agricultural Research Service (ARS), United States Department of Agriculture (USDA), Albany, California, United States of America; University of Wisconsin-Milwaukee, United States of America

## Abstract

**Background:**

In the U.S., tomatoes have become the most implicated vehicle for produce-associated Salmonellosis with 12 outbreaks since 1998. Although unconfirmed, trace backs suggest pre-harvest contamination with *Salmonella enterica*. Routes of tomato crop contamination by *S. enterica* in the absence of direct artificial inoculation have not been investigated.

**Methodology/Principal Findings:**

This work examined the role of contaminated soil, the potential for crop debris to act as inoculum from one crop to the next, and any interaction between the seedbourne plant pathogen *Xanthomonas campestris* pv. vesicatoria and *S. enterica* on tomato plants. Our results show *S. enterica* can survive for up to six weeks in fallow soil with the ability to contaminate tomato plants. We found *S. enterica* can contaminate a subsequent crop via crop debris; however a fallow period between crop incorporation and subsequent seeding can affect contamination patterns. Throughout these studies, populations of *S. enterica* declined over time and there was no bacterial growth in either the phyllosphere or rhizoplane. The presence of *X. campestris* pv. vesicatoria on co-colonized tomato plants had no effect on the incidence of *S. enterica* tomato phyllosphere contamination. However, growth of *S. enterica* in the tomato phyllosphere occurred on co-colonized plants in the absence of plant disease.

**Conclusions/Significance:**

*S. enterica* contaminated soil can lead to contamination of the tomato phyllosphere. A six week lag period between soil contamination and tomato seeding did not deter subsequent crop contamination. In the absence of plant disease, presence of the bacterial plant pathogen, *X. campestris* pv. vesicatoria was beneficial to *S. enterica* allowing multiplication of the human pathogen population. Any event leading to soil contamination with *S. enterica* could pose a public health risk with subsequent tomato production, especially in areas prone to bacterial spot disease.

## Introduction

In recent years, tomatoes have been one of the most common vehicles of produce-associated Salmonellosis. In the United States, there have been 12 outbreaks caused by *Salmonella enterica* related to tomato consumption since 1998 [1], [2]. The tomato contamination appears to originate from the fields where the tomatoes were grown and/or the packing sheds [3]. However, the route of contamination remains indefinable, although probable suspects exist: water, soil, animal waste, and insects [4].

It has been shown in a hydroponic system that *S. enterica* can contaminate entire tomato plants following direct root inoculations [5]. Furthermore, tomato fruits can be contaminated with *S. enterica* following direct flower inoculations [6]. These routes of inoculation probably do not reflect the natural contamination route of field grown tomatoes. However, the colonization or contamination of tomato plants in the absence of direct artificial inoculation has not been investigated thoroughly.

*S. enterica* has been shown to contaminate carrots, radish, lettuce and parsley in field studies, following treatments with contaminated manure compost or irrigation water; however, these crops were also directly contaminated [7], [8]. We undertook this study to determine the ability of *S. enterica* to colonize or contaminate tomato plants via indirect contamination. We chose to limit our study to the contamination of tomato plants and survival of *S. enterica* in the phyllosphere and rhizoplane of plants preceding flower set. Other studies have addressed the contamination of tomato fruit following contamination of flowers and fruit in contact with contaminated soil [6], [9]; however, examination of plant contamination by *S. enterica* through indirect inoculation paths prior to this developmental stage was nonexistent. We chose two paths for indirect contamination, soil contamination from an irrigation event with contaminated water or contaminated crop debris from a previous crop. Furthermore, we investigated the role of a plant pathogen in the survival and population size of *S. enterica* in both the phyllosphere and rhizoplane.

## Materials and Methods

### Bacterial Strains, Plasmid, and Growth Media

*S. enterica* serovar Baildon strain 05x-02123 [2] and serovar Enteritidis strain 99A-23 (California Health Department, July 2005 outbreak) are clinical isolates from tomato-related outbreaks and were used as a 1:1 mixture. These strains were chosen due to their involvement in tomato Salmonellosis outbreaks. Although the strains were not differentiated during population enumeration, both strains were used in this study to ensure inclusion of biological variability which may exist among *S. enterica* serovars during soil survival or plant contamination and colonization. The plant pathogen, *Xanthomonas campestris* pathovar vesicatoria was isolated from the cultivated tomato (*Lycopersicon esculentum* Mill.), diseased with bacterial spot, field grown in 2004 (Davis, CA). All bacteria were grown on Luria-Bertani (LB) media and *S. enterica* populations were enumerated on *Salmonella Shigella* (SS) media (Difco/BBL; Sparks, MD). Kanamycin (Sigma, St. Louis, MO) was incorporated into all media at 40 mg/liter. Plasmid pKT-kan, in which a 131 bp *nptII* promoter fragment from Tn*5* was fused to the *gfp* gene of plasmid pPROBE-KT, is a stable, broad-host-range vector that confers kanamycin resistance and green fluorescent protein (*gfp*) expression [10]. Plasmid pKT-kan was transformed into both strains of *S. enterica*; this plasmid has been shown to have no affect on survival and growth of *S. enterica* [11].

### Plant Assays

*S. enterica* was inoculated directly into Supersoil (Rod McLellan Co., San Mateo, CA), an enriched potting soil (total nitrogen 0.14%, available P_2_O_5_ 0.09%, soluble potash K_2_O 0.02%, total iron 0.25%, Canadian sphagnum peat moss, ground fir bark, compost, and sand in a proprietary blend) at a pH of 5.5–6.5. Overnight bacterial streak cultures grown at 37°C were suspended in sterile water with a sterile swab to an OD_600 nm_ of 0.2 (∼10^8^ CFU/ml) and bacteria were mixed 1:1 and diluted to the necessary concentration. Soil (approximately 215 g) was either autoclaved or not (non-sterile), placed in 10.5 cm^2^ pots, and irrigated once with 25 ml of *S. enterica* suspensions (10^3^, 10^5^, or 10^7^ CFU/ml). Pots were kept in a controlled-environment growth chamber under a day and night cycle of 12 h, during which the day temperature was 26°C and the night temperature was 18°C. Humidity was constant at 75%. Controls were soil irrigated with sterile water. Tomato seeds (cultivar Moneymaker; Tomato Bob, Hilliard, OH) were surface sanitized with 3% calcium hypochlorite as described previously [12] and soaked in sterile water in Petri plates for 1 h to remove any remaining sanitizer. Seeds were sown in *S. enterica* contaminated soil 24 h post soil inoculation. Pots were returned to the growth chamber immediately following seed sowing. Pots were irrigated (approximately 25 ml sterile water) every 48 h or 24 h, once tomato plants were four weeks old.

Soil was assayed for *S. enterica* 24 h post-inoculation and once weekly for long term survival assays. Soil samples were placed in tared 15 ml conical tubes (approximately 3 g samples), weighed, and 10 ml of sterile water was added. The suspension was vortexed on high for 1 min. Serial dilutions of the suspension were plated on SS agar with kanamycin, plates were incubated at 42°C (to select for the growth of *S. enterica* over indigenous soil bacteria) for 24 h, and *S. enterica* populations were enumerated. Black colonies were confirmed as the inoculated *S. enterica* strains by confirmation of gfp expression under UV illumination of the plates.

Plants were removed from the soil whole and soil was gently removed from the roots by shaking. Using a sterile razor blade, tomato plants were cut in two; separating the above the soil (phyllosphere) plant parts from that which was below the soil line (rhizoplane). At the seedling stage (approximately 10 to 13 days-old), plant parts were put into separate tared microfuge tubes, weighed, and 1 ml of sterile water was added. The tubes were vortexed for 1 min, serial dilutions of the suspension were made and aliquots were plated on SS agar. LB broth (with kanamycin) was added to the plant samples and incubated overnight at 37°C, with shaking at 150 rpm. If no colonies grew on the original SS agar-Kan, one microliter loop of the enrichment was streaked on SS agar-Kan and incubated at 42°C for 24 h to confirm the presence of *S. enterica* in the plant samples.

### Debris studies

Soil was inoculated with *S. enterica* and seeds sown as described above. Plants were grown for 30 days and then the entire plant was cut into approximately 2.5 cm pieces, including the roots. Soil was removed from the roots by shaking. Plant debris was mixed with non-sterile soil at a ratio of 1:4 by weight, placed in pots, and kept in a growth chamber, same conditions as described above. Twenty-four hours or one week later, seeds for the second crop were treated with calcium hypochlorite and sown as described above. The second crop was assayed for *S. enterica* populations at the seedling and three to five leaf stages. Controls were seeds sown in non-sterile soil with debris from plants grown in soil irrigated with sterile water instead of *S. enterica*. Pots were irrigated (approximately 25 ml sterile water) every 48 h.

### Plant pathogen assays

To investigate whether the presence of a bacterial tomato plant pathogen effects the *S. enterica* population, soil was autoclaved and inoculated with the *S. enterica* cocktail as described above. An overnight streak culture of *X. campestris* pv. vesicatoria was suspended in sterile water with a sterile swab to an OD_600 nm_ of 0.2 (∼10^8^ CFU/ml) in 20 ml. Following calcium hypochlorite treatment and 1 h soaking in sterile water, tomato seeds were soaked in the *X. campestris* pv. vesicatoria suspension for 1 h, continuously shaking at 40 rpm. Seeds were then sown in the *S. enterica* contaminated soil as described above. Populations of *X. campestris* pv. vesicatoria on seed were determined immediately proceeding planting and 24 and 48 h post sowing. To enumerate the bacteria, individual seeds were placed in 1 ml of sterile water and vortexed for 1 min, serial dilutions of the suspension were made, aliquots were plated on LB agar, and plates were incubated overnight at 28C. Seeds not inoculated with *X. campestris* pv. vesicatoria were soaked in sterile water and otherwise treated similarly to the others.

Tomato plants were assayed for *S. enterica* populations at the seedling stage, three to five leaf stage (approximately 25 to 28 days-old), and pre-bloom (approximately 35 to 42 days-old). Plants were sampled as described above with the exception of three to five leaf and pre-bloom stage plant parts were placed into tared 50 ml conical tubes, weighed, and 10 ml of sterile water was added.

### Experimental design and statistics

For each study, pots were seeded with six to eight seeds per pot. For the long term survival assays, two pots were seeded for each week (6 time periods) starting one week after soil contamination. At the seedling stage, all plants were sampled per time period. The experiment was repeated twice. For the plant debris studies, five pots were seeded for each fallow period. Approximately, half of the plants were sampled at each plant growth stage, seedling and 3–5 leaf stage. The experiment was repeated twice. For the plant pathogen studies, three pots were used for each *S. enterica* inoculum level and with or without plant pathogen seed inoculation. Approximately half of the plants were sampled at each plant growth stage. The experiment was repeated twice. To determine whether the average populations or incidence of *S. enterica* differed between treatments or over time, populations were log transformed and unpaired t-tests with a Welch correction were performed using GraphPad Instat (version 3.06, GraphPad Software, Inc., San Diego, CA).

## Results

### *S. enterica* survived six weeks in fallow soil with the capacity to contaminate tomato plants

**Figure 1:**
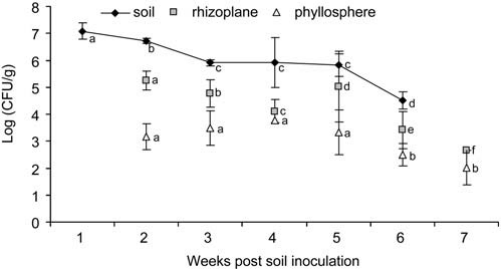
Average *Salmonella enterica* populations in soil and on leaves, stems, and roots of tomato. Tomato seeds were sown at weekly intervals in *S. enterica* irrigated soil and *S. enterica* populations were enumerated at the seedling stage. Phyllosphere and rhizoplane populations were from seedlings planted one week before sampling. Averages from two experiments were calculated from log transformed data and error bars represent standard deviations. T-tests were preformed to determine average population changes over time for each type of sample. Different letters represent significant population changes (p<0.03).

To test the longevity of the *S. enterica* population in soil and whether these cells could contaminate plants, soil was irrigated with *S. enterica* contaminated water, seeds were sown at weekly intervals, and plants were examined for *S. enterica*. *S. enterica* was able to survive in fallow soil and remained capable of plant attachment and contamination at least six weeks following introduction to the soil by irrigation (Fig. 1). *S. enterica* soil populations declined for three weeks, stabilized for week three to five, and declined again for the sixth week. At six weeks fallow, *S. enterica* soil populations were approximately three logs lower than their initial levels. Overall, *S. enterica* populations in the rhizoplane were significantly smaller with each weekly sampling, except between the fourth and fifth week. *S. enterica* populations in the phyllosphere were stable for five weeks and smaller each week thereafter. *S. enterica* was not recovered from control plants.

### Contaminated plant debris can serve as inoculum to subsequent crops

To determine the role crop debris may play in the persistence of *S. enterica* in fields used for continuous tomato cropping, a contaminated tomato crop was produced by irrigating soil with *S. enterica*, direct seeding tomato into the *S. enterica* contaminated soil, and allowing the crop to develop for 30 days. The crop was mulched and mixed with soil, and tomato seeds (second crop) were sown in the crop debris soil mixture either 24 h or 7 d later. *S. enterica* was recovered from the phyllosphere and rhizoplane of the second crop when seeds were sown 24 h following plant debris incorporation (Table 1). At the three to five leaf stage, the *S. enterica* population in the phyllosphere was below the level of enumeration and required enrichment for detection. *S. enterica* was not recovered from the phyllosphere of the second crop whose seeds were sown in soil which had lain fallow for seven days. *S. enterica* rhizoplane populations remained below 100 CFU/g for the duration of the study. *S. enterica* was not recovered from control plants.

**Table 1:** Incidence of *Salmonella enterica* contamination following sowing in non-sterile soil mixed with contaminated plant debris from a previous tomato crop.

	1 day fallow*		7 days fallow	
	Phyllosphere	Rhizoplane	Phyllosphere	Rhizoplane
Seedling	2/18+	14/18	0/20	8/20
3–5 Leaf	2/15	15/15	0/12	11/12

* Tomato seeds were sown one or seven days following crop incorporation.

+ Number of *S. enterica* positive samples/number of samples tested. These data are combined from two experiments.

### Plant pathogen causes higher *S. enterica* populations

To investigate whether the presence of a bacterial tomato plant pathogen affects *S. enterica* in association with tomato, seeds were inoculated with *X. campestris* pv. vesicatoria, causal agent of bacterial spot of tomato, and sown in *S. enterica* contaminated autoclaved soil. Immediately proceeding sowing, *X. campestris* pv. vesicatoria treated seeds contained 4.8×10^4^±4.2×10^3^ CFU/seed and decreased to 1.2×10^4^±1.4×10^3^ CFU/seed after 24 h in soil. At the seedling, 3–5 leaves, and pre-bloom stages, all rhizoplane samples, regardless of the presence of *X. campestris* pv. vesicatoria, were colonized by *S. enterica*. There was no statistical difference between the incidence of *S. enterica* contaminated tomato plants, rhizoplane or phyllosphere, with and without *X. campestris* pv. vesicatoria plant colonization.

**Figure 2:**
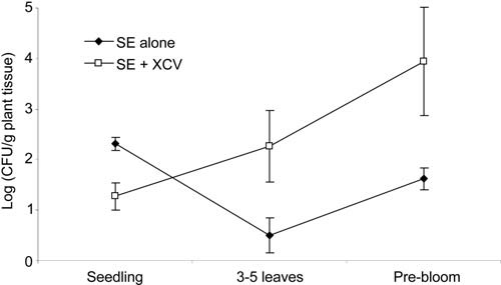
Average *Salmonella enterica* populations in the tomato phyllosphere. S. enterica populations are from plants with (white square) or without (black diamond) inoculation of tomato seed with *Xanthomonas campestris* pv. vesicatoria. Autoclaved soil was irrigated with *S. enterica* 24 h prior to seed sowing. Averages from two experiments were calculated from log transformed data and error bars represent standard deviations.

To determine whether *S. enterica* soil contamination levels could affect the subsequent contamination of the tomato plant, tomato seeds with and without *X. campestris* pv. vesicatoria were seeded in either soil with an initial high (∼10^5^ CFU/ml) or low (∼10^3^ CFU/ml) *S. enterica* inoculum level. There was no difference between the *S. enterica* populations on plants with or without *X. campestris* pv. vesicatoria seeded in the high inoculum soil. However, the *S. enterica* populations on plants seeded in the low inoculum soil differed at each plant development stage between those with or without *X. campestris* pv. vesicatoria plant colonization (Fig. 2; p≤0.005). At the seedling stage, *S. enterica* populations on plants not co-colonized by *X. campestris* pv. vesicatoria, were significantly higher than those of the co-colonized plants. At the three to five leaves and pre-bloom stages, *S. enterica* populations were significantly higher on those plants which were co-colonized by *X. campestris* pv. vesicatoria.

## Discussion

Identifying the source of pre-harvest *S. enterica* contamination of fresh produce has been elusive, yet identification and risk assessment of each contamination route is necessary to control foodborne illness caused by consumption of raw produce in the absence of an available “kill-step” between farm and fork. Irrigation water has been implicated as a source of fresh produce contamination and subsequent human disease [13]. Castillo and colleagues isolated serovars of *S. enterica* that were the same as the strains which caused three large Salmonellosis outbreaks associated with cantaloupe consumption [14]. These serovars were found in the irrigation water of farms that produced contaminated melons. Drip irrigation is used for melon production in this area; thus, if melon contamination occurred from the irrigation water it was likely via the soil. These reports led us to examine the ability of *S. enterica* to contaminate tomato plants from irrigation water via soil contamination, in light of the many Salmonellosis outbreaks caused by contaminated fresh tomatoes.

Our results reveal the ability of *S. enterica* to attach to and contaminate both the phyllosphere and rhizoplane of tomato plants via soil following irrigation with contaminated water. Furthermore, contamination of the phyllosphere and rhizoplane occurred for the duration of our six week study, suggesting contaminated soil has the potential to inoculate seeds and subsequently crops weeks after a soil contamination event. Survival of *S. enterica* in fallow soil has been reported up to five weeks [15], [16]; however, the ability to subsequently contaminate agricultural crops has not been addressed. One may assume *S. enterica* has the capacity to contaminate plants seeded in contaminated soil; however, there are no reported studies to this effect, nor has the affect of a lag period between contamination event and crop seeding been examined. We recovered *S. enterica* populations in fallow contaminated soil weekly up to six weeks. Furthermore, these cells were capable of attaching to and contaminating the rhizoplane and phyllosphere of tomato plants from seeds sown in these soils. These results suggest any event, i.e., flooding, raw manure or contaminated compost applications, or excretion by infected or carrier animals, which introduce *S. enterica* into the soil, could lead to subsequent crop contamination; though time may pass between the contamination event and planting.

Soil survival by enteric pathogens, i.e., *S. enterica*, appears dependent on many factors including soil type and cropping system [7], [8], [17]. In our study and fresh produce fields studied by others, *S. enterica* populations are not stable and decline over time. Thus, factors which can increase *S. enterica* persistence are a public health concern. Because plant pathogens can utilize crop debris to colonize subsequent crops [18], [19], we examined the capacity of crop debris to act as an inoculum source for *S. enterica* to a tomato crop. Our results reveal *S. enterica* contaminated crop debris can lead to contamination of a subsequent crop. The success of subsequent crop colonization appears dependent on the fallow period between crop incorporation and subsequent seeding, as seen by the absence of *S. enterica* recovery from the phyllosphere of plants sown following a one week fallow period between crops. Replanting fields shortly following harvest of a previous crop is a common practice, e.g., lettuce in the Salinas Valley of California [18] and tomato transplants in south Florida. Fields known to have produced crops contaminated with *S. enterica* may benefit from extended fallow periods between crops. Switching to produce that require cooking before eating would also reduce the risk of foodborne illness.

It is well established that the incidence of *S. enterica* on fresh produce is significantly higher on diseased or injured produce [20], [21]. Surprisingly, in our study, the incidence of *S. enterica* contamination was not significantly different between the plants co-colonized with and without the plant pathogen *X. campestris* pv. vesicatoria, in the absence of disease. There was no difference between the rhizoplane *S. enterica* populations of tomato co-colonized or not by the plant pathogen. Whether *S. enterica* can benefit from the presence of growing roots, since roots are known to release nutrients into the immediate soil available to bacteria [22], remains to be studied further.

When *S. enterica* populations in the contaminated soil were low, the presence of *X. campestris* pv. vesicatoria had an interesting effect on subsequent colonization of the tomato phyllosphere. At the seedling stage, *S. enterica* populations in the phyllosphere were lower on the co-colonized plants compared to plants without *X. campestris* pv. vesicatoria. We hypothesize that the lower *S. enterica* populations were due to some advantage of *X. campestris* pv. vesicatoria for plant colonization and thus, excluded or outcompeted *S. enterica* at the seedling stage. At the three to five leaves and pre-bloom stages, *S. enterica* populations of the co-colonized plants grew in the phyllosphere. Thus, our study shows that in the absence of a plant pathogen, *S. enterica* could not grow and subsequently colonize the tomato plant. These results suggest a beneficial interaction for *S. enterica* between the human and plant pathogen prior to plant disease development. Whether the plant pathogen had proceeded to breakdown the plant tissue at a microscopic level was not investigated, but can not be ignored. However, *X. campestris* pv. vesicatoria populations did not reach populations high enough to cause disease (data not shown) [23]. Several noncompeting hypotheses may explain the advantage afforded *S. enterica* on tomato plants co-colonized by *X. campestris* pv. vesicatoria. *S. enterica* might utilize *X. campestris* pv. vesicatoria by joining its aggregates or biofilm on the leaf surface, which *S. enterica* may not produce for itself in the tomato phyllosphere. *X. campestris* pv. vesicatoria may overcome the innate immune plant response, similar to *Pseudomonas syringae* pv. tomato [24]; thus, allowing both *X. campestris* pv. vesicatoria and *S. enterica* to enter leaf tissue thru open stomata. Further research is needed to support or correct these hypotheses governing the interaction of *S. enterica* and bacterial plant pathogens on plants in the absence of plant disease.

## References

[1] CDC (2005). Outbreaks of *Salmonella* infections associated with eating Roma tomatoes–United States and Canada, 2004.. MMWR Morb Mortal Wkly Rep.

[2] Cummings K, Barrett E, Mohle-Boetani JC, Brooks JT, Farrar J (2001). A multistate outbreak of *Salmonella enterica* serotype Baildon associated with domestic raw tomatoes.. Emerg Infect Dis.

[3] CDC (2007). Multistate outbreaks of *Salmonella* infections associated with raw tomatoes eaten in restaurants—United States, 2005–2006.. MMWR Morb Mortal Wkly Rep.

[4] Brandl MT (2006). Fitness of human enteric pathogens on plants and implications for food safety.. Annu Rev Phytopathol.

[5] Guo X, van Iersel MW, Chen J, Brackett RE, Beuchat LR (2002). Evidence of association of Salmonellae with tomato plants grown hydroponically in inoculated nutrient solution.. Appl Environ Microbiol.

[6] Guo X, Chen J, Brackett RE, Beuchat LR (2001). Survival of Salmonellae on and in tomato plants from the time of inoculation at flowering and early stages of fruit development through fruit ripening.. Appl Environ Microbiol.

[7] Islam M, Morgan J, Doyle MP, Phatak SC, Millner P (2004). Fate of *Salmonella enterica* serovar Typhimurium on carrots and radishes grown in fields treated with contaminated manure composts or irrigation water.. Appl Environ Microbiol.

[8] Islam M, Morgan J, Doyle MP, Phatak SC, Millner P (2004). Persistence of *Salmonella enterica* serovar Typhimurium on lettuce and parsley and in soils on which they were grown in fields treated with contaminated manure composts or irrigation water.. Foodborne Pathog Dis.

[9] Guo X, Chen J, Brackett RE, Beuchat LR (2002). Survival of *Salmonella* on tomatoes stored at high relative humidity, in soil, and on tomatoes in contact with soil.. J Food Prot.

[10] Miller WG, Leveau JH, Lindow SE (2000). Improved gfp and *ina*Z broad-host-range promoter-probe vectors.. Mol Plant Microbe Interact.

[11] Charkowski AO, Barak JD, Sarreal CZ, Mandrell RE (2002). Differences in growth of *Salmonella enterica* and *Escherichia coli* O157:H7 on alfalfa sprouts.. Appl Environ Microbiol.

[12] Barak JD, Gorski L, Naraghi-Arani P, Charkowski AO (2005). *Salmonella enterica* virulence genes are required for bacterial attachment to plant tissue.. Appl Environ Microbiol.

[13] Garcia-Villanova Ruiz B, Espinar AC, Bolanos Carmona MJ (1987). A comparative study of strains of *Salmonella* isolated from irrigation waters, vegetables and human infections.. Epidem Inf.

[14] Castillo A, Mercado I, Lucia LM, Martinez-Ruiz Y, Ponce de Leon J (2004). *Salmonella* contamination during production of cantaloupe: a binational study.. J Food Prot.

[15] Jensen AN, Dalsgaard A, Stockmarr A, Nielsen EM, Baggesen DL (2006). Survival and transmission of *Salmonella enterica* serovar typhimurium in an outdoor organic pig farming environment.. Appl Environ Microbiol.

[16] Nicholson FA, Groves SJ, Chambers BJ (2005). Pathogen survival during livestock manure storage and following land application.. Bioresour Technol.

[17] Natvig EE, Ingham SC, Ingham BH, Cooperband LR, Roper TR (2002). *Salmonella enterica* serovar typhimurium and *Escherichia coli* contamination of root and leaf vegetables grown in soils with incorporated bovine manure.. Appl Environ Microbiol.

[18] Barak JD, Koike ST, Gilbertson RL (2001). Role of crop debris and weeds in the epidemiology of bacterial leaf spot of lettuce in California.. Plant Dis.

[19] Gilbertson RL, Rand RE, Hagedorn DJ (1990). Survival of *Xanthomonas campestris* pv. phaseoli and pectolytic strains of *X. campestris* in bean debris.. Plant Dis.

[20] Wells JM, Butterfield JE (1997). *Salmonella* contamination associated with bacterial soft rot of fresh fruits and vegetables in the marketplace.. Plant Dis.

[21] Wells JM, Butterfield JE (1999). Incidence of *Salmonella* on fresh fruits and vegetables affected by fungal rots or physical injury.. Plant Dis.

[22] Jaeger CH, Lindow SE, Miller W, Clark E, Firestone MK (1999). Mapping of sugar and amino acid availability in soil around roots with bacterial sensors of sucrose and tryptophan.. Appl Environ Microbiol.

[23] Hirano SS, Upper CD (2000). Bacteria in the leaf ecosystem with emphasis on *Pseudomonas syringae*-a pathogen, ice nucleus, and epiphyte.. Microbiol Mol Biol Rev.

[24] Melotto M, Underwood W, Koczan J, Nomura K, He SY (2006). Plant stomata function in innate immunity against bacterial invasion.. Cell.

